# The Marine Triterpene Stellettin B Triggers Mitochondrial-to-Nuclear Translocation of AIF/EndoG and Reverses Epithelial-Mesenchymal Transition to Inhibit Oral Cancer Progression

**DOI:** 10.7150/ijms.134063

**Published:** 2026-07-22

**Authors:** Peng-Yu Chen, Pin-Chen Kuo, Yi-Lung Ling, Yen-Yun Wang, Shyng-Shiou F. Yuan, Jyh-Horng Sheu, Wan-Chi Tsai

**Affiliations:** 1 School of Dentistry, College of Dental Medicine, Kaohsiung Medical University, Kaohsiung 807, Taiwan.; 2 Department of Medical Laboratory Science and Biotechnology, College of Health Sciences, Kaohsiung Medical University, Kaohsiung 807, Taiwan.; 3 Drug Development and Value Creation Research Center, Kaohsiung Medical University, Kaohsiung 807, Taiwan.; 4 Department of Medical Research, Kaohsiung Medical University Hospital, Kaohsiung 807, Taiwan.; 5 Graduate Institute of Medicine, College of Medicine, Kaohsiung Medical University, Kaohsiung 807, Taiwan.; 6 Translational Research Center, Kaohsiung Medical University Hospital, Kaohsiung 807, Taiwan.; 7 Department of Obstetrics and Gynecology, Kaohsiung Medical University Hospital, Kaohsiung 807, Taiwan.; 8 Department of Marine Biotechnology and Resources, National Sun Yat-sen University, Kaohsiung 804, Taiwan.; 9 Department of Laboratory Medicine, Kaohsiung Medical University Hospital, Kaohsiung 807, Taiwan.

## Abstract

Oral squamous cell carcinoma (OSCC) is associated with aggressive clinical behavior and poor outcomes. In this study, we investigated the anticancer efficacy and underlying mechanisms of Stellettin B, an isomalabaricane triterpene isolated from the marine sponge *Jaspis stellifera,* in OSCC cells. Our results demonstrate that Stellettin B significantly inhibited the proliferation of HSC-3 and OC-2 cells while sparing normal oral keratinocytes. Mechanistically, Stellettin B triggers a predominantly caspase-independent apoptotic program, evidenced by the pronounced mitochondrial-to-nuclear translocation of apoptosis-inducing factor (AIF) and endonuclease G (EndoG) following DNA damage, whereas classical caspase activation functions as a dispensable, secondary event. Furthermore, Stellettin B suppressed migration and invasion by reversing epithelial-mesenchymal transition (EMT), characterized by E-cadherin upregulation and downregulation of Vimentin, Snail, Slug, and β-catenin. Transcriptomic profiling further revealed significant suppression of mTORC1 signaling and EDIL3 expression. In conclusion, these findings demonstrate that Stellettin B exerts multimodal antitumor activity in OSCC and highlight its therapeutic potential as a marine-derived anticancer agent.

## Introduction

Head and neck squamous cell carcinoma (HNSCC) remains the sixth most common cancer globally, with over 870,000 new cases and 450,000 deaths recorded annually 1. Oral squamous cell carcinoma (OSCC) is the most predominant subtype of HNSCC and has shown a rising incidence in recent years. Despite advancements in diagnostic techniques and multidisciplinary treatment approaches, the five-year survival rate for patients diagnosed at advanced stages remains poor, often falling below 50%. This disappointing clinical outcome underscores the urgent need for novel therapeutic strategies beyond conventional surgery, radiotherapy, and chemotherapy 2, 3.

Natural products serve as an indispensable reservoir for anticancer drug development due to their remarkable structural diversity and potent biological activities. Currently, more than 60% of approved anticancer agents are derived from or inspired by natural sources, with the marine environment emerging as a particularly prolific source of unique secondary metabolites with high clinical potential 4, 5. Although several marine-derived compounds have achieved translational success in oncology 6, 7, their therapeutic utility has yet to be fully explored in the specific context of oral malignancies, highlighting a distinct gap in marine-based drug discovery for OSCC.

Stellettin B, a triterpene isolated from the marine sponge *Jaspis stellifera*, exhibits broad-spectrum antineoplastic activity. In various non-oral cancers (e.g., lung, leukemia, liver, and glioblastoma), it triggers cell cycle arrest, apoptosis, and autophagy, and suppresses metastasis by blocking oncogenic pathways like PI3K/Akt/mTOR and MAPK 8-13. Although recently noted to induce OSCC cell death via endoplasmic reticulum stress and mitochondrial pathways 14, whether Stellettin B effectively targets the high migratory and invasive propensity of oral cancer remains completely unknown. Given that local invasion and regional lymph node metastasis are the primary drivers of poor prognosis and treatment failure in OSCC, characterizing the anti- metastatic dimensions and underlying molecular cascades of Stellettin B is of paramount clinical importance.

Consequently, the present study was designed to systematically evaluate the cytotoxic and anti-metastatic effects of Stellettin B on OSCC cells. To bridge the identified mechanistic gaps, we investigated the dual functionality of this compound, specifically focusing on the engagement of a caspase-independent (AIF/EndoG) apoptotic pathway, while exploring its potential correlative link to cellular motility suppression through the modulation of the EDIL3/EMT axis.

## Materials and Methods

### Cell culture and reagents

Human OSCC cell lines (OC-2, HSC-3, SCC-9, OEC-M1, CAL-27, and SAS) and primary human oral keratinocytes (HOK) were utilized in this study. HSC-3 cells were maintained in MEM supplemented with 10% fetal bovine serum (FBS), 1% penicillin/streptomycin (P/S), 1% sodium pyruvate (SP), and 1% non-essential amino acids (NEAA). OC-2 and OEC-M1 cells were cultured in RPMI-1640, while CAL-27, SAS, and SCC-9 cells were grown in DMEM/F12, both supplemented with 10% FBS, 1% P/S, and 1% SP. HOK were cultured in specialized oral keratinocyte medium containing growth supplements (OKGS) and 1% P/S. All cultures were incubated at 37°C in a humidified atmosphere with 5% CO₂. Cell lines were purchased from the Japanese Collection of Research Bioresources (JCRB), ATCC, Bioresource Collection and Research Center (Hsinchu, Taiwan), Cell Line Service (Germany), or ScienCell Research Laboratories (USA).

### Natural product preparation

Stellettin B (MW: 462.6), an isomalabaricane-type triterpene characterized by its highly oxygenated framework and unique stereochemistry, was isolated from the marine sponge *Jaspis stellifera* 15. The compound was generously provided by Professor Jyh-Horng Sheu (National Sun Yat-sen University). For experimental use, a 100 mM stock solution was prepared in dimethyl sulfoxide (DMSO) and stored at -20 °C.

### Cell viability and proliferation assays

Cytotoxicity and proliferation were assessed using MTT, BrdU, and EdU assays as previously described 16. For standard viability tracking, HSC-3 cells seeded in 96-well plates were treated with Stellettin B for 24-48 h. To assess caspase dependency, cells were allowed to adhere overnight and pretreated with 20 μM of the pan-caspase inhibitor Z-VAD-FMK (TargetMol, Cat. No. T7020) for 1 h, followed by a 48 h co-treatment with 200 nM Stellettin B. Following treatments, cells were incubated with MTT reagent, and absorbance was measured at 595 nm. Cell proliferation was quantified using a BrdU ELISA kit (Merck) following 48 h of Stellettin B exposure. For EdU incorporation, the Click-iT™ EdU imaging kit (Invitrogen) was used, with nuclei counterstained by DAPI and visualized via confocal microscopy (Olympus FV1000).

### Apoptosis and cell cycle analysis

Apoptotic populations were quantified using the Annexin V-FITC/PI Detection Kit (Strong Biotech, Taiwan) and analyzed by flow cytometry (BD FACSLyric™). Active Caspase-3 was detected using a FITC-conjugated apoptosis kit (BD Pharmingen™). For cell cycle distribution, cells were stained with propidium iodide (PI; 40 μg/ml) and analyzed by flow cytometry as previously established 16.

### Comet assay for DNA damage

DNA strand breaks were evaluated using the comet assay under neutral and alkaline conditions. Stellettin B-treated cells were embedded in low-melting agarose on precoated slides. Following lysis (2.5 M NaCl, 100 mM EDTA, 10 mM Tris, 1% Triton X-100, pH 10), electrophoresis was performed at 30 V (15 min) for alkaline conditions or 21 V (45 min) for neutral conditions. Slides were subsequently neutralized, dehydrated, and stained with PI.

### Western blotting

Cells were treated with Stellettin B, lysed in RIPA buffer containing protease inhibitors, and subjected to SDS-PAGE and PVDF transfer. Membranes were blocked with 5% non-fat milk and incubated overnight at 4°C with primary antibodies: β-actin (1:5000, sc-47778, Santa Cruz), β-catenin (1:1000, 8480S, Cell Signaling Technology), cleaved caspase-3 (1:1000, 9661S, Cell Signaling Technology), caspase-7 (1:1000, 9492S, Cell Signaling Technology), caspase-8 (1:1000, 9746S, Cell Signaling Technology), caspase-9 (1:1000, 9502S, Cell Signaling Technology), E-cadherin (1:1000, sc-7870, Santa Cruz), Endonuclease G (1:1000, 4969S, Cell Signaling Technology), phospho-Histone H2A.X (1:1000, 9718S, Cell Signaling Technology), Slug (1:1000, 9585S, CST), Snail (1:2000, 3895S, Cell Signaling Technology), vimentin (1:1000, sc-6260, Santa Cruz), and EDIL3 (1:750, sc-293337, Santa Cruz). After washing, membranes were incubated with HRP-conjugated secondary antibodies (Santa Cruz) for 1 h at room temperature. Protein bands were detected with ECL reagent (Amersham) and visualized using a chemiluminescence imaging system. Band intensities were quantified using ImageJ software.

### Wound healing, migration, and invasion assays

For the wound healing assay, confluent HSC-3 monolayers in 6-well plates (2 ×10^5^ cells/well) were scratched with a sterile 200-μL pipette tip, washed with PBS, and incubated in fresh serum-free medium containing Stellettin B (0, 25, and 50 nM). Migration distances were quantified using ImageJ from inverted microscope images captured at 0 and 12 h post-wounding. Cell migration and invasion were evaluated using 24-well Transwell inserts (8.0-μm pore size; 353097, Corning); with inserts precoated with 100 μL of Matrigel (1:8 dilution) for the invasion assay only. Briefly, 1 ×10^5^ HSC-3 cells in 200 μL of serum-free medium ± Stellettin B were seeded into the upper chambers, with 500 μL of 10% FBS medium in the lower chambers. After incubation (12 h for migration; 24 h for invasion), non-migrated/invaded cells were removed from the upper surface. Lower-surface adherent cells were fixed with 4% paraformaldehyde, stained with 0.1% crystal violet, and quantified by counting five random fields per well at 200× magnification.

### Immunofluorescence staining

To detect mitochondrial-to-nuclear translocation of AIF and EndoG, fixed cells were incubated with anti-AIF (1:250, sc-13116, Santa Cruz) or anti-EndoG (1:250, ab9647, Abcam) antibodies, followed by fluorophore-conjugated secondary antibodies (Invitrogen). Nuclei were counterstained with DAPI, and images were captured using an Olympus FV1000 confocal microscope.

### RNA sequencing-based exploratory screening and gene set enrichment analysis (GSEA)

For preliminary transcriptomic profiling and candidate gene screening, HSC-3 cells were treated with Stellettin B (200 nM) or vehicle control (0.1% DMSO) for 48 h. Total RNA was extracted using TRIzol reagent (Invitrogen), and an exploratory, single-run library preparation/sequencing workflow was conducted by Biotools Co., Ltd. (Taiwan) to identify high-priority candidate pathways and downstream targets. Gene Set Enrichment Analysis (GSEA) was performed using MSigDB Hallmark gene sets (h.all.v2023.1.Hs). Normalized enrichment scores (NES), nominal p-values, and false discovery rate (FDR q-values) were calculated, and pathways with FDR q-value < 0.25 were considered significantly enriched, following standard GSEA guidelines.

### RT-qPCR and RNA quality control

RNA was purified using the Direct-zol™ RNA MiniPrep kit (Zymo Research) with on-column DNase I digestion. RNA purity (A260/A280 > 1.8) was verified using a Take3 Micro-Volume Plate on a Synergy microplate reader with Gen5 software (BioTek Instruments, USA). Complementary DNA was synthesized using the SensiFAST™ cDNA Synthesis Kit (Bioline, UK). Quantitative PCR was performed on an Applied Biosystems 7500 System (Thermo Fisher Scientific, USA) using SYBR Green. Target gene expression was normalized to GAPDH and calculated using the 2^⁻ΔΔCT^ method. The primers for EDIL3 (112 bp) were: 5'-GCGAATGGAACTTCTTGGCTGTG-3' (forward) and 5'-GAGCGTTCTGAAGATGCTGGAG-3' (reverse).

### Statistical analysis

Data are presented as mean ± standard deviation (SD) from at least three independent experiments. Statistical significance was determined using Student's *t*-test, with *p* < 0.05 considered significant (*p < 0.05, **p < 0.01, ***p < 0.001).

## Results

### Stellettin B selectively inhibits the viability and proliferation of OSCC cells

**Figure 1:**
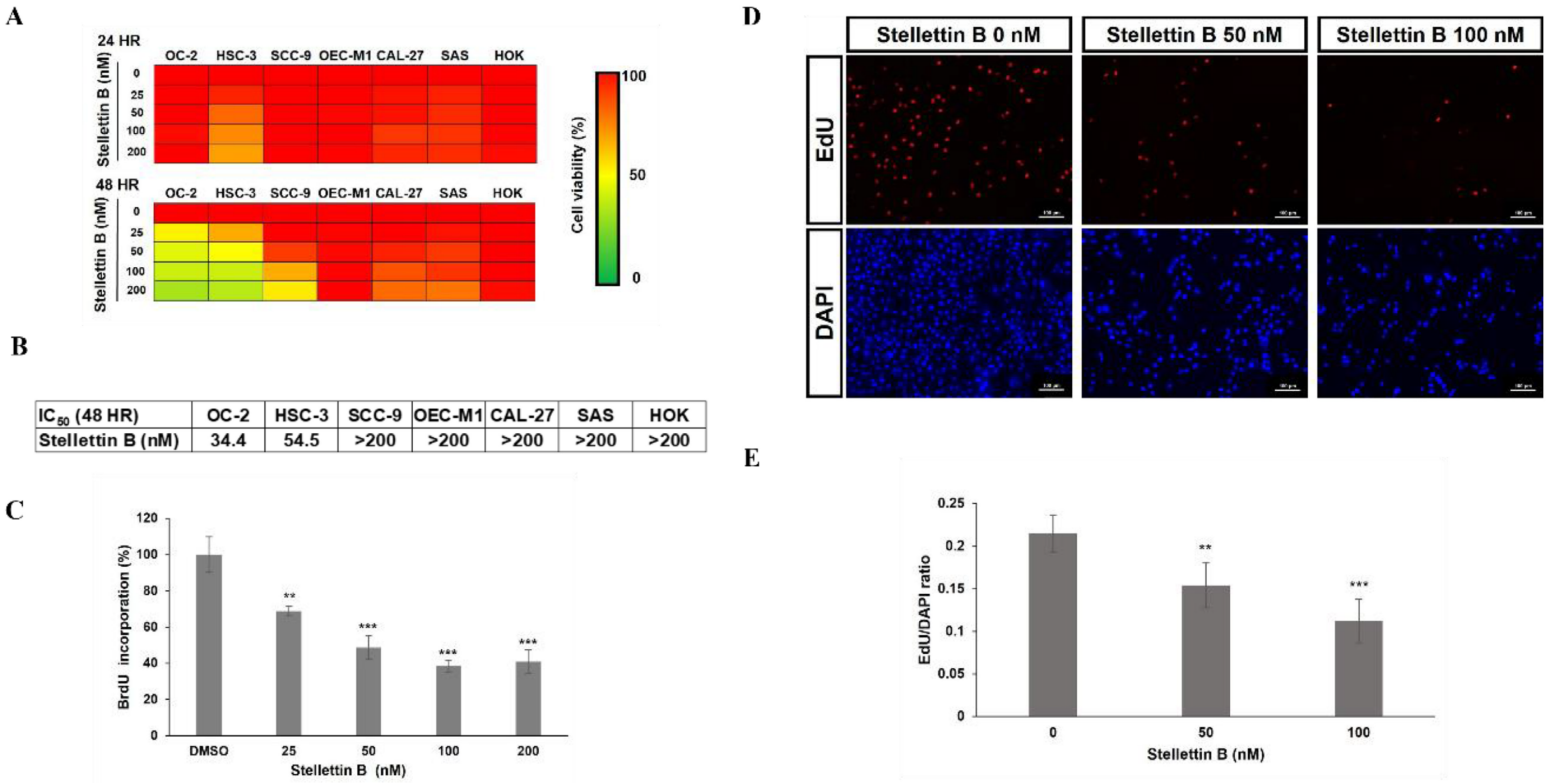
** Stellettin B selectively inhibits the viability and proliferation of OSCC cells.** (A) Dose-dependent effect of Stellettin B on the viability of various OSCC cell lines (OC-2, HSC-3, SCC-9, OEC-M1, CAL-27, and SAS) after 24 and 48 h treatment. (B) Comparative viability of HSC-3, OC-2, and normal oral keratinocytes (HOK) after Stellettin B treatment, demonstrating selective cytotoxicity. (C-E) Suppression of DNA synthesis by Stellettin B in OC-2 and HSC-3cells, as assessed by BrdU incorporation and EdU imaging (Scale bar = 100 μm). Data are presented as mean ± SD of three independent experiments (**p < 0.01, ***p < 0.001 vs. control).

To evaluate the anticancer potential of Stellettin B, we first examined its effect on the viability of several human oral squamous cell carcinoma (OSCC) cell lines, including OC-2, HSC-3, SCC-9, OEC-M1, CAL-27, and SAS. The MTT assay results demonstrated that Stellettin B significantly reduced the cell viability of all tested OSCC lines in a dose-dependent manner (Figure 1A). Specifically, OC-2 and HSC-3 cells exhibited marked sensitivity to the treatment, prompting their selection for further mechanistic studies. The IC₅₀ values for Stellettin B at 48 hours were determined to be 54.5 nM for HSC-3 and 34.4 nM for OC-2 cells. Importantly, Stellettin B showed minimal cytotoxicity toward normal human oral keratinocytes (HOK), with cell viability remaining at approximately 98% even at concentrations as high as 200 nM (Figure 1B). Consequently, the IC_50_ for HOK was determined to be > 200 nM. To quantify this preferential cytotoxicity, the Selectivity Index (SI) was calculated (SI = IC_50_ of HOK / IC_50_ of OSCC cells). The SI values for Stellettin B were > 5.8 for OC-2 and > 3.7 for HSC-3 cells, respectively, exceeding the standard baseline (SI > 2-3) for high tumor selectivity and indicating a high therapeutic index toward malignant cells. To further corroborate these findings, we assessed the impact of Stellettin B on cell proliferation using BrdU and EdU incorporation assays. The results indicated that treatment with Stellettin B significantly suppressed DNA synthesis in a dose-dependent manner in both HSC-3 and OC-2 cells (Figure 1C-E). These data collectively demonstrate that Stellettin B exerts potent anti-proliferative effects specifically on OSCC cells without compromising the viability of normal oral keratinocytes.

### Stellettin B induces apoptosis in HSC-3 cells

**Figure 2:**
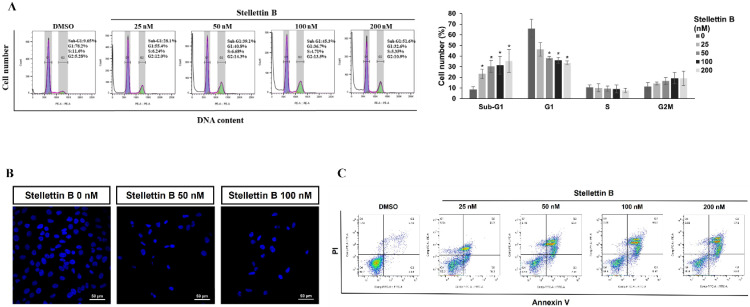
** Stellettin B induces cell apoptosis in HSC-3 cells.** (A) Flow cytometric analysis of cell cycle distribution in HSC-3 cells treated with indicated concentrations of Stellettin B for 48 h. The continuous expansion of the sub-G1 population is highlighted. Statistical quantification of cell cycle distribution (Sub-G1, G1, S, and G2/M phases) presented as a bar graph. Data are expressed as mean ± SD from three independent experiments. *p < 0.05 indicates a statistically significant difference compared with the control (0 nM) group. (B) Representative fluorescence images of DAPI-stained nuclei showing morphological features of apoptosis (nuclear condensation and fragmentation) after Stellettin B treatment. (C) Quantification of early and late apoptosis using Annexin V-FITC/PI double staining and flow cytometry.

To further investigate the mechanisms underlying the growth-inhibitory effects of Stellettin B, we analyzed its impact on cell cycle distribution in HSC-3 cells. Flow cytometric analysis revealed that treatment with Stellettin B led to a significant and dose-dependent increase in the sub-G1 population, which is indicative of DNA fragmentation and apoptosis. Specifically, following 48 hours of treatment with 200 nM Stellettin B, the sub-G1 population increased from a baseline of 8.7% in the control group to approximately 35.4% following 48 hours of treatment with 200 nM Stellettin B (Figure 2A). Concurrently, this apoptotic accumulation was accompanied by a dose-dependent decrease in the G1 phase population, dropping from 65.9% to 33.7%, whereas changes in the G2/M population lacked statistical significance. These dynamics demonstrate that the primary cytotoxic impact of Stellettin B is the induction of massive apoptosis, characterized by robust sub-G1 expansion, rather than a definitive cell cycle phase arrest (Figure 2A). The pro-apoptotic effect of Stellettin B was further characterized using DAPI nuclear staining and Annexin V-FITC/PI double staining. DAPI staining showed characteristic morphological changes of apoptosis, such as nuclear condensation and fragmentation, in HSC-3 cells treated with Stellettin B (Figure 2B). Furthermore, Annexin V/PI flow cytometry demonstrated a significant, dose-dependent rise in both early and late apoptotic cell populations. After 48 hours of exposure to higher concentrations of Stellettin B, approximately 60% of the cells underwent apoptosis (Figure 2C). These findings confirm that the reduction in cell viability observed earlier is primarily driven by the induction of programmed cell death and cell cycle arrest by Stellettin B.

### Stellettin B triggers a predominantly caspase-independent apoptotic program accompanied by DNA damage

**Figure 3:**
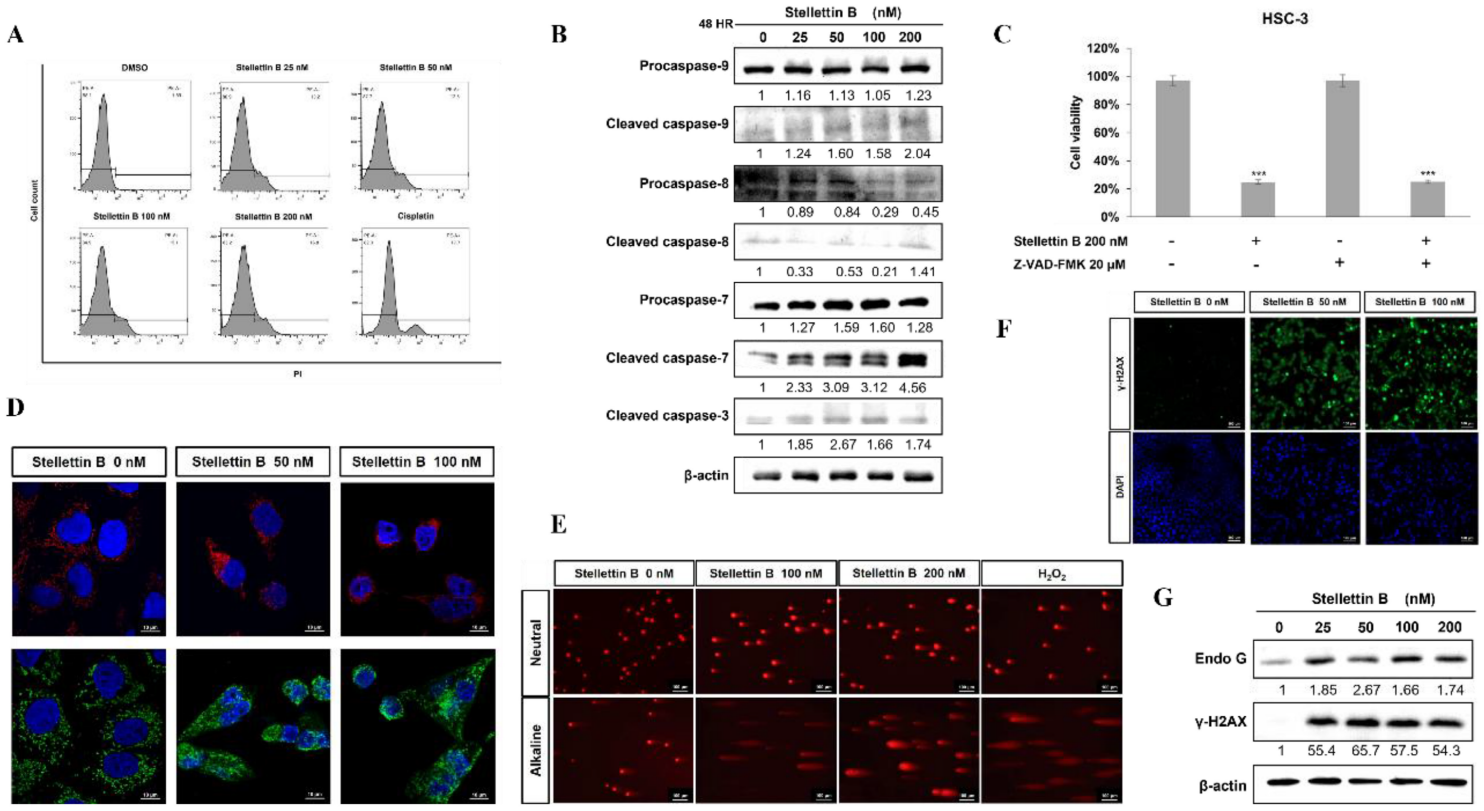
** Stellettin B triggers cell death predominantly through a caspase-independent apoptotic pathway driven by DNA damage.** HSC-3 cells were treated with indicated concentrations of Stellettin B for the designated time points as described in Materials and Methods. (A) Flow cytometric showing the percentage of active caspase-3-positive cells. (B) Western blot profiles of intrinsic (cleaved caspase-9) and extrinsic (cleaved caspase-8), and executioner (cleaved caspase-3/-7) apoptotic markers. (C) MTT cell viability assay of HSC-3 cells pretreated with the pan-caspase inhibitor Z-VAD-FMK (20 μM) for 1 h followed by Stellettin B (200 nM) co-treatment for 48 h. The pharmacological blockade of caspase activity failed to rescue the loss of cell viability. (D) Immunofluorescence imaging demonstrating the mitochondrial-to-nuclear translocation of AIF (green) and EndoG (red) in HSC-3 cells; nuclei were counterstained with DAPI (blue) (Scale bar = 20 μm). (E) Neutral and alkaline comet assays indicating the induction of DNA strand breaks. (F, G) Evaluation of DNA double-strand breaks via immunofluorescence tracking of γ-H2AX foci formation (green; nuclei counterstained with DAPI, blue) and corresponding Western blot analysis of EndoG and γ-H2AX protein expression. Data are expressed as mean ± SD from three independent biological replicates. ***p < 0.001 compared with the vehicle control (DMSO).

To elucidate the molecular machinery driving Stellettin B-induced cell death, we evaluated both canonical caspase-dependent cascades and non-canonical caspase-independent pathways. Flow cytometric analysis measuring active caspase-3 levels revealed that Stellettin B treatment failed to induce substantial caspase-3 activation in HSC-3 cells, with only a baseline percentage of cells showing positivity across all tested concentrations, which was notably weaker than the positive control Cisplatin (Figure 3A). To further dissect this on a molecular level, Western blot analysis was performed (Figure 3B). While Stellettin B treatment provoked a dose-dependent accumulation of cleaved caspase-7 and a moderate increase in cleaved caspase-9, the executioner cleaved caspase-3 remained minimally detectable and lacked a robust dose-responsive pattern. To definitively determine whether caspase activation is functionally required for Stellettin B-mediated cytotoxicity, we performed a rescue assay using the pan-caspase inhibitor Z-VAD-FMK (Figure 3C). Co-treatment with Z-VAD-FMK (20 µM) did not significantly attenuate the massive reduction in HSC-3 cell viability induced by 200 nM Stellettin B, establishing that classical caspase activation is not the primary driver of execution.

We therefore investigated the involvement of caspase-independent factors, specifically apoptosis-inducing factor (AIF) and endonuclease G (EndoG). Immunofluorescence staining clearly demonstrated the mitochondrial-to-nuclear translocation of both AIF and EndoG in HSC-3 cells treated with Stellettin B, providing strong evidence for the activation of this pathway (Figure 3D). Crucially, many literatures establish that the mitochondrial-to-nuclear translocation of AIF and EndoG is an autonomous, sufficient executioner mechanism for caspase-independent cell death 17-19. Upon nuclear entry, AIF directly induces large-scale DNA fragmentation and chromatin condensation 17, while EndoG autonomously executes nucleosomal DNA cleavage 18, 19. Since nuclear translocation of these factors is often associated with DNA damage, we performed comet assays and analyzed γ-H2AX expression. Stellettin B treatment led to significant DNA strand breaks under both neutral and alkaline conditions (Figure 3E). Furthermore, a dose-dependent increase in γ-H2AX protein levels and its nuclear foci formation were observed, confirming the induction of DNA double-strand breaks (Figure 3F, G). Taken together, these findings demonstrate that Stellettin B-induced OSCC apoptosis is predominantly driven by a caspase-independent AIF/EndoG translocation pathway operating downstream of unrepairable DNA damage.

### Stellettin B inhibits OSCC cell motility and invasion by reversing the epithelial-mesenchymal transition

**Figure 4:**
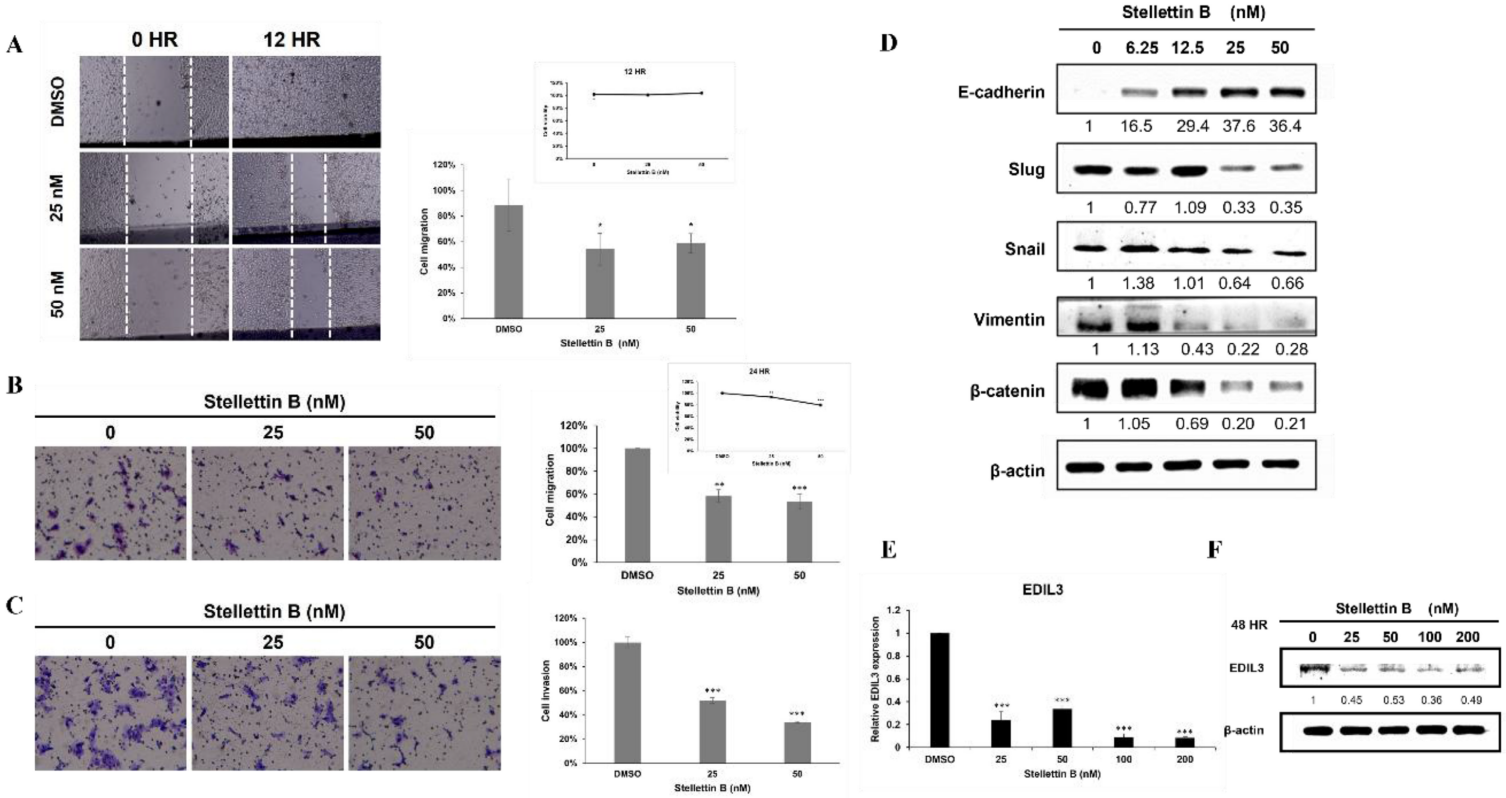
** Stellettin B modulates EDIL3 expression and reverses the EMT phenotype to suppress OSCC cell motility.** HSC-3 cells were treated with indicated concentrations of Stellettin B for the designated time points. (A) Wound healing assay illustrating the inhibition of lateral cell migration and wound closure capacity after 12 h of treatment; embedded line graphs (insets) indicate the corresponding cell viability. (B) Transwell migration assay showing a reduction in the number of migratory cells following a 12 h exposure. (C) Transwell invasion assay demonstrating marked suppression of invasive cell capabilities through Matrigel after 24 h of treatment. (D) Western blot analysis of EMT-associated markers and regulatory factors following 48 h of treatment. (E, F) RT-qPCR (112 bp product) and Western blot analyses validating the dose-dependent suppression of EDIL3 expression at both mRNA and protein levels, respectively, following 48 h of treatment. Data are expressed as mean ± SD from three independent experiments. *p < 0.05, **p < 0.01, ***p < 0.001 compared with the vehicle control (DMSO).

To evaluate the anti-metastatic potential of Stellettin B, we performed wound healing and Transwell migration/invasion assays using HSC-3 cells. To exclude confounding cytotoxic effects, cell viability was monitored at the exact time points and doses of each functional assay and embedded as inset line graphs within the quantification panels (Figure 4A-C). At 12 hours post-treatment, the inset line graph in Figure 4A demonstrates that cell viability remained completely unaffected, maintaining approximately 101% and 104% viability at 25 and 50 nM of Stellettin B, respectively. Concurrently, the wound healing assay revealed that treatment with Stellettin B significantly suppressed wound closure within this non-toxic 12-hour window (Figure 4A). Similarly, within a highly viable 24 h window (> 80% viability), Transwell migration (Figure 4B) and invasion (Figure 4C) capacities were markedly and dose-dependently reduced. Because these assay timeframes (12-24 h) are shorter than the doubling time of OSCC cells (> 24 h), these results explicitly demonstrate that the suppressed cell motility is driven by the active inhibition of migration and invasion rather than passive growth suppression or cell death.

Since cellular motility in OSCC is frequently governed by the epithelial-mesenchymal transition (EMT), we examined relevant markers via Western blotting. Stellettin B treatment led to a dose-dependent upregulation of the epithelial marker E-cadherin, alongside a significant reduction in mesenchymal markers (Vimentin) and EMT-inducing transcription factors (Snail, Slug, and β-catenin) (Figure 4D). To unearth the underlying molecular drivers, exploratory transcriptomic profiling was employed, identifying EDIL3 (epidermal growth factor-like repeats and discoidin I-like domains 3) as a prominently downregulated candidate gene. Subsequent rigorous validation via RT-qPCR and Western blot analysis confirmed that Stellettin B consistently suppressed EDIL3 expression at both the mRNA and protein levels (Figure 4E, F). Collectively, these data suggest that Stellettin B impairs OSCC migratory and invasive capabilities by reversing the EMT process, potentially through the downstream targeting of the motility-associated factor EDIL3.

## Discussion

Oral squamous cell carcinoma (OSCC) is characterized by its high propensity for local invasion and regional lymph node metastasis, which significantly compromises patient survival. In this study, we demonstrated that Stellettin B exerts potent anticancer activity through a coordinated, multi-layered mechanism involving apoptosis induction, suppression of oncogenic signaling, and reversal of epithelial-mesenchymal transition (EMT).

A key mechanistic finding of this research is that Stellettin B triggers apoptosis predominantly through a caspase-independent pathway. While initial markers suggested the involvement of canonical apoptotic signaling, our detailed functional and Western blot analyses revealed a profile that deviated from the classical dose-dependent caspase activation. Specifically, the lack of a linear increase in cleaved caspase-3 and the failure of the pan-caspase inhibitor Z-VAD-FMK to rescue cell viability, despite significant sub-G1 accumulation, establishes that caspase-dependent signaling is merely a secondary, dispensable event rather than the primary executioner. This is supported by the prominent mitochondrial-to-nuclear translocation of AIF and EndoG, alongside marked DNA fragmentation. Although the explicit temporal sequence was not dynamically monitored here, extensive literature establishes that severe DNA damage (marked by $\gamma$-H2AX accumulation) acts as the critical upstream trigger. This genomic stress activates nuclear sensors (e.g., PARP-1 or ATM), which compromise mitochondrial membrane permeability and drive the nuclear translocation of AIF and EndoG to execute apoptosis 20-22. The ability of Stellettin B to predominantly activate the AIF/EndoG axis provides a strategic advantage in overcoming cancers that have developed resistance to traditional caspase-dependent therapies.

To further elucidate the molecular landscape underlying the effects of Stellettin B, preliminary transcriptomic profiling and Gene Set Enrichment Analysis (GSEA) were performed as an exploratory, hypothesis-generating screen. A prominent finding was the significant suppression of the mTORC1 signaling hallmark gene set following Stellettin B treatment (Supplementary Figure S1). mTORC1 is a central regulator of cellular growth, metabolism, and survival in head and neck cancers 23. Inhibition of mTORC1 typically suppresses downstream anabolic signaling via effectors such as S6K1 and 4E-BP1, thereby increasing apoptotic vulnerability in malignant cells 24. Interestingly, emerging evidence suggests that mTORC1 signaling may exhibit a close correlative link to β-catenin-mediated transcriptional activity, thereby contributing to partial EMT states and tumor progression. 25, 26. Consistent with this notion, our data demonstrate a marked reduction in β-catenin expression following Stellettin B treatment. While the comprehensive upstream-to-downstream epistatic network remains to be fully delineated, this concurrent alteration of mTORC1 and β-catenin signaling provides a compelling candidate pathway that potentially correlates cell death induction with the modulation of tumor cell plasticity.

A significant highlight of our study is the identification of EDIL3 (epidermal growth factor-like repeats and discoidin I-like domains 3) as a prominent and validated downstream target of Stellettin B. Screened initially as a top candidate among the differentially expressed genes (DEGs) in our exploratory RNA-seq analysis, EDIL3 transcripts were found to be markedly downregulated following treatment. This finding was consistently validated by independent testing via RT-qPCR and protein quantification, which demonstrated a dose-dependent suppression of EDIL3 at both the mRNA and translational levels (Figure 4E, F). As an extracellular matrix (ECM)-associated protein, EDIL3 has been established as a driver of EMT, cell motility, and angiogenesis in various malignancies, including breast, prostate, and lung cancers 27, 28. In OSCC, its suppression may disrupt integrin-mediated signaling and downstream transcriptional programs governing EMT. Consistently, Stellettin B treatment resulted in the upregulation of E-cadherin and downregulation of mesenchymal markers, including vimentin, Snail, and Slug, indicating a reversal of the EMT phenotype.

Collectively, these transcriptomic insights suggest that Stellettin B coordinates a multifaceted anti-tumor response in OSCC cells: it triggers an initial signaling cascade linked to mTORC1 suppression that facilitates caspase-independent apoptosis, while simultaneously driving the robust downregulation of EDIL3, which may correlate with the attenuation of β-catenin signaling and the subsequent reversal of the EMT phenotype. Guided by this framework, our findings support a preliminary mechanistic model in which Stellettin B exerts anticancer effects through interconnected alterations of cellular processes: (i) induction of multimodal apoptosis driven primarily by the AIF/EndoG-mediated pathway, (ii) concurrent modulation of potential candidate oncogenic pathways, including mTORC1/β-catenin signaling, and (iii) inhibition of EMT and cell motility, which is observed to be accompanied by the downstream targeting of EDIL3. This integrative phenotype not only highlights the therapeutic potential of Stellettin B but also underscores the importance of further exploring these interconnected signaling pathways in OSCC.

Despite these promising findings, several limitations should be acknowledged. First, the present study was conducted exclusively using* in vitro* cell models; consequently, *in vivo* validation in xenograft or orthotopic models will be essential to confirm therapeutic efficacy, pharmacokinetics, and safety. Second, while EDIL3 downregulation strongly correlated with EMT reversal, explicit genetic interventional experiments—such as establishing stable EDIL3 overexpression rescue clones or knockdown lines to confirm a direct causal axis governing Vimentin, Snail, Slug, and β-catenin—were beyond the scope of this initial study. Third, as our transcriptomic analysis was employed strictly for preliminary candidate screening, the detailed functional cascades and direct regulatory lines linking exploratory targets like mTORC1 suppression to AIF/EndoG-mediated apoptosis or EMT dynamics remain to be independently mapped in future investigations. Addressing these avenues will further clarify the translational potential of Stellettin B.

In conclusion, Stellettin B is a multifunctional marine-derived compound that suppresses OSCC progression through coordinated regulation of apoptosis, oncogenic signaling, and metastatic phenotypes. These findings provide a strong foundation for further preclinical development of Stellettin B as a potential therapeutic agent for oral cancer.

## Supplementary Material

Supplementary figure.

## Data Availability

Data available within the article or its Supplementary Materials.
