# Supplementary material for: The Marine Triterpene Stellettin B Triggers Mitochondrial-to-Nuclear Translocation of AIF/EndoG and Reverses Epithelial-Mesenchymal Transition to Inhibit Oral Cancer Progression
Source: Int J Med Sci. 2026 Jul 22;23(8):2758–66. doi: 10.7150/ijms.134063 (PMC13411358; doi:10.7150/ijms.134063)
Supplement: Supplementary file 1 — Supplementary figure. [file ijmsv23p2758s1.pdf]

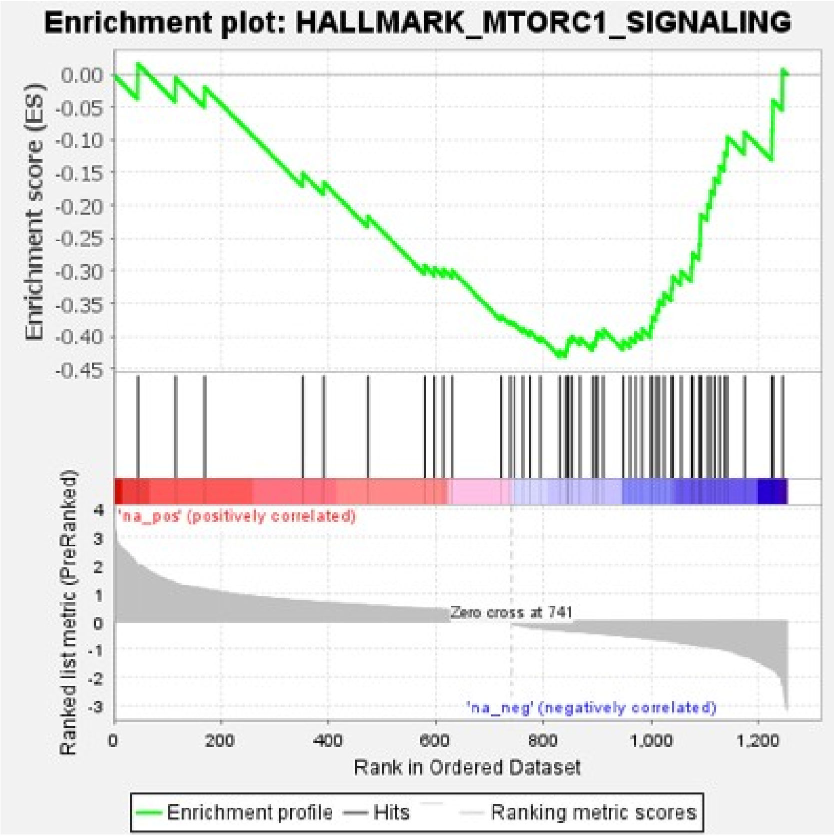

Supplementary Figure S1. Molecular validation of EDIL3 downregulation and EMT gene set enrichment.

Gene Set Enrichment Analysis (GSEA) plot demonstrating the significant downregulation of the mTORC1 signaling hallmark gene set in Stellettin B-treated HSC-3 cells. The Normalized Enrichment Score (NES) and False Discovery Rate (FDR) *q*-value indicate a robust suppression of this central survival and metabolic pathway.
